# Differential Gene Expression in the Oxyntic and Pyloric Mucosa of the Young Pig

**DOI:** 10.1371/journal.pone.0111447

**Published:** 2014-10-30

**Authors:** Michela Colombo, Davide Priori, Paolo Trevisi, Paolo Bosi

**Affiliations:** Dipartimento di Scienze e Tecnologie Agro-alimentari, Università di Bologna, Bologna, Italy; University of California, Los Angeles, United States of America

## Abstract

The stomach is often considered a single compartment, although morphological differences among specific areas are well known. Oxyntic mucosa (OXY) and pyloric mucosa (PYL, in other species called antral mucosa) are primarily equipped for acid secretion and gastrin production, respectively, while it is not yet clear how the remainder of genes expressed differs in these areas. Here, the differential gene expression between OXY and PYL mucosa was assessed in seven starter pigs. Total RNA expression was analyzed by whole genome Affymetrix Porcine Gene 1.1_ST array strips. Exploratory functional analysis of gene expression values was done by Gene Set Enrichment Analysis, comparing OXY and PYL. Normalized enrichment scores (NESs) were calculated for each gene (statistical significance defined when False Discovery Rate % <25 and *P*-values of NES<0.05). Expression values were selected for a set of 44 genes and the effect of point of gastric sample was tested by analysis of variance with the procedure for repeated measures. In OXY, HYDROGEN ION TRANSMEMBRANE TRANSPORTER ACTIVITY gene set was the most enriched set compared to PYL, including the two genes for H^+^/K^+^-ATPase. Pathways related to mitochondrial activity and feeding behavior were also enriched (primarily cholecystokinin receptors and ghrelin). Aquaporin 4 was the top-ranking gene. In PYL, two gene sets were enriched compared with OXY: LYMPHOCYTE ACTIVATION and LIPID RAFT, a gene set involved in cholesterol-rich microdomains of the plasma membrane. The single most differentially expressed genes were gastrin and secretoglobin 1A, member 1, presumably located in the epithelial line, to inactivate inflammatory mediators. Several genes related to mucosal integrity, immune response, detoxification and epithelium renewal were also enriched in PYL (*P*<0.05). The data indicate that there is significant differential gene expression between OXY of the young pig and PYL and further functional studies are needed to confirm their physiological importance.

## Introduction

The stomach essentially is devoted to the preparation of the bolus for the best digestion conditions in the downstream digestive tracts. Neural, hormonal, paracrine signals resulting from luminal content sensing (chemicals and nutrients, xenobiota components), are integrated in the stomach [1] to adjust the intake, passage rate and metabolism in collaboration with the intestine.

In the pig, oxyntic glands are found in the cardia gland and fundic gland regions (**OXY**), while antral-type mucous glands are found in the pyloric gland region (**PYL**).

This is reflected by their main functions of acid secretion and gastrin secretion, respectively. Differential expressions of numerous gene groups highlight the different specializations of the gastric mucosa compared with the small and large intestines [2]. However, it is not documented if these differences are unique to the whole stomach or two functional mucosal compartments.

Additional knowledge about the compartmentalization between OXY and PYL would help to identify markers of gastric areas [3] and provide models to investigate the developmental process of the gastric mucosa in normal or specific conditions, such as during weaning.

The development of specific physiological functions of the stomach is relevant for the young pig to rapidly adapt to post-weaning diets and also to control the gastro-intestinal microbiota using acid secretion or other defenses. Several feeding strategies have been proposed to improve the health of piglets [4] and improved knowledge of the differential gene expression in the two specialized gastric mucosal areas would help to improve feeding practices and provide further markers in addition to those already used [5]–[8].

Our aim is to assess the differential gene expression between OXY and PYL in young pigs.

## Materials and Methods

The procedures carried out on the pigs were conducted in compliance with Italian laws on experimental animals and were approved by the Ethic-Scientific Committee for Experiments on Animals of the University of Bologna (Permit number: ARIC- 47357).

### Animals and sample collection

Seven crossbred (Large White × Landrace) male weaned pigs (5–6 weeks of age, 11.1 kg body weight, on average) were individually housed in cages and a standard post-weaning feed for five days. Then, after the morning meal, pigs were slaughtered by intracardiac injection (Tanax, 0.5 mL/kg body weight; Intervet Italia, Peschiera Borromeo, Italy), after being anaesthetized with sodium thiopental (10 mg/kg body weight). For each subject, the stomach was removed, opened along the greater curvature and washed in ice-cold PBS, and two samples with transmural sections were collected respectively for OXY in the great curvature between the cardiac gland region and for PYL in the pyloric gland region close to the pyloric sphincter. Samples were immediately frozen in liquid nitrogen and stored at −80°C until use.

### RNA Isolation, Microarray Processing, Quality Control

Total RNA was isolated from oxyntic and PYL collected from each subject, according to Qiagen RNeasy Midi Kit protocol (Qiagen, Hilden, Germany). To reduce the viscosity of the lysate, specimens (50 to 100 mg) were homogenized directly in the buffer RTL containing guanidine thiocyanate. All the other procedures were in agreement with the manufacturer protocol. Purity and integrity evaluation was assessed just before analysis by Agilent Bioanalyzer 2100. Total RNA was hybridized on Affymetrix Porcine Gene 1.1 ST array strips. Hybridized arrays were scanned on a GeneAtlas imaging station (Affymetrix, Santa Clara, CA, USA). Performance quality tests of the arrays including the labelling, hybridization, scanning and background signals by a Robust Multichip Analysis were performed on the CEL files using Affymetrix Expression Console. The intensity records were log2-transformed. Transcript data have been submitted to the National Center for Biotechnology Information's Gene Expression Omnibus (NCBI GEO) with GEO accession number GSE57620.

### Gene quantification by real-time RT-PCR

Samples were validated by the quantification of the expression of H+/K+ Atpase α (***ATP4A*** *)*; gastrin *(* ***GAST*** *)*; ghrelin/obestatin prepropeptide (***GHRL***); polymeric immunoglobulin receptor (***PIGR*** *)* genes by real-time quantitative PCR (RT-qPCR). 1 µg of total RNA was reverse-transcribed using the ImProm-II Reverse Transcription System (Promega), for all the genes, primers were designed based on a specific porcine nucleic acid sequence (Gen-Bank database) using Primer 3 version 0.4.0 (http://frodo.wi.mit.edu/primer3/). The primers sequences, amplicon length and annealing/extension temperatures are given in Table 1.

**Table 1:** Primers information and RT-qPCR conditions used in the trial.

Gene^1^	NCBI accession number	Oligo sequence (5′→3′)		Amplicon length	Annealing T
*ATP4A*	M22724	Forward Reverse	GCATATGAGAAGGCCGAGAG TGGCCGTGAAGTAGTCAGTG	151 pb	57°C
*GAST*	NM_001004036	Forward Reverse	GACTCTGCGCCTATGTCCTG GCTCTTTGCCCCTGTTGG	133 bp	60°C
*GHRL*	NM213807	Forward Reverse	GAACAGAGGTGGCTGGTCTC ACAGGGGAGACAAGGAAAGG	202 pb	62°C
*PIGR*	NM_214159.1	Forward Reverse	AGCCAACCTCACCAACTTCC CTGCTAATGCCCAGACCAC	105 bp	62°C
*HMBS*	DQ845174	Forward Reverse	AGGATGGGCAACTCTACCTG GATGGTGGCCTGCATAGTCT	83 bp	62°C
*RPL4*	DQ845176	Forward Reverse	CAAGAGTAACTACAACCTTC GAACTCTACGATGAATCTTC	122 bp	60°C

1 *ATP4A*, H+/K+ Atpase α; *GAST*, gastrin; *GHRL*, ghrelin/obestatin prepropeptide; *PIGR*, polymeric immunoglobulin receptor; *HMBS*, hydroxymethylbilane synthase; *RPL4*, ribosomal protein L4.

The RT-qPCR reaction was performed in a LightCycler Real-Time PCR Systems (Roche Applied Science) by a shuttle PCR (2 steps) following the procedure described by Trevisi et al. [9]. The expression data were normalized by geometric mean of the expression of the two housekeeping genes: hydroxymethylbilane synthase (***HMBS2***) and ribosomal protein L4 (***RPL4***). Primers and amplification conditions for the housekeeping genes are reported in Table 1.

### Pathway Analysis and other statistics

Affymetrix Trascripts IDs, each one in general characterized by several exonic sequences, were associated with 13,406 human gene names based on the *Sus scrofa* Ensembl database (release 69, www.ensembl.org). For the processed gene expression values, exploratory functional analysis was done with Gene Set Enrichment Analysis using the C5.BP catalog of the gene sets (based on Gene Ontology) from Molecular Signatures Database v3.1(http://www.broadinstitute.org/gsea/msigdb/Index.jsp), comparing OXY with PYL. Normalized enrichment scores (**NES**s) were calculated for each gene set and statistical significance was defined when the False Discovery Rate % was <25 and the *P*-values of the NES were <0.05, as suggested by the program. Enrichment score *P-*values were estimated using a gene set-based permutation test procedure.

From microarray analysis, expression values were obtained for a preselected set of 45 genes, identified on the basis of the literature [2] and our previous observations. The effect of the kind of gastric mucosa (OXY or PYL) was tested on these data by analysis of variance with the SAS GLM (SAS Inst. Inc., Cary, NC, USA) procedure for repeated measures (each pig).

## Results

In OXY, a total of 18 gene sets were significantly enriched compared with PYL (Table 2). HYDROGEN ION TRANSMEMBRANE TRANSPORTER ACTIVITY was the gene set most differentially enriched, which includes the two genes for H^+^/K^+^-ATPase, fundamental enzyme for acid secretion; pathways related to mitochondrial activity and feeding behavior were also enriched (the last involving primarily cholecystokinin receptors, GHRL and the anorexigenic neuropeptide W). Aquaporin 4, water-selective channel protein present in the plasma membranes, was the top-ranking gene.

**Table 2:** Gene sets enriched in oxyntic and pyloric mucosae of young pigs.

Name	Size^1^	NES^2^	FDR^3^ q-value
*Oxyntic mucosa*			
HYDROGEN_ION_TRANSMEMBRANE_ TRANSPORTER_ACTIVITY	20	2.172	0.002
MITOCHONDRION	268	2.149	0.002
MITOCHONDRIAL_MEMBRANE_PART	39	2.094	0.002
MITOCHONDRIAL_RESPIRATORY_CHAIN	18	2.029	0.009
MITOCHONDRIAL_INNER_MEMBRANE	50	2.029	0.008
CELLULAR_RESPIRATION	16	1.990	0.014
MONOVALENT_INORGANIC_CATION_ TRANSMEMBRANE_TRANSPORTER_ACTIVITY	24	1.981	0.014
ORGANELLE_INNER_MEMBRANE	56	1.956	0.019
FEEDING_BEHAVIOR	21	1.955	0.018
MITOCHONDRIAL_PART	104	1.896	0.040
INORGANIC_CATION_TRANSMEMBRANE_ TRANSPORTER_ACTIVITY	44	1.877	0.050
MITOCHONDRIAL_MEMBRANE	64	1.847	0.071
ENERGY_DERIVATION_BY_OXIDATION_OF_ ORGANIC_COMPOUNDS	31	1.823	0.088
MITOCHONDRIAL_ENVELOPE	73	1.797	0.108
CHEMOKINE_ACTIVITY	28	1.760	0.150
CHEMOKINE_RECEPTOR_BINDING	29	1.740	0.176
HYDROLASE_ACTIVITY_ACTING_ON_CARBON_NITROGEN_NOT_PEPTIDEBONDSIN_LINEAR_ AMIDES	17	1.692	0.246
LIGAND_DEPENDENT_NUCLEAR_RECEPTOR_ ACTIVITY	22	1.679	0.248
ATPASE_ACTIVITY_COUPLED_TO_ TRANSMEMBRANE_MOVEMENT_OF_IONS	21	1.660	0.247
*Pyloric mucosa*			
LYMPHOCYTE_ACTIVATION	49	−1.770	0.236
LIPID_ RAFT	24	−1.760	0.221

1 Number of genes in the set.

2 Normalized enrichment score.

3 False discovery rate.

In PYL, only two gene sets were significantly enriched compared with OXY: LYMPHOCYTE ACTIVATION, with interleukin 7 ranking first, and LIPID RAFT, a gene set involved in specialized membrane domains composed mainly of cholesterol and sphingolipids. The single gene most differentially expressed was *GAST*, the peptide hormone produced in pylorus by G cells. The second most differentially expressed gene was ***SCGB1A1***, secretoglobin, family 1A, member 1, presumably located in the epithelial line, to inactivate inflammatory mediators.

Among the set of pre-selected genes, 16 genes were more expressed in OXY compared with PYL (Table 3). These genes were related to acid secretion and pH homeostasis (ATP4A; anion exchanger 2) and were ion and water channels (potassium voltage-gated channel, isk-related family, member 2– ***KCNE2***; potassium inwardly rectifying channel, subfamily J, member 13 and member 15– **KCNJ13** and ***KCNJ15***; chloride intracellular channel 6– ***CLIC6***; aquaporin 4), endocrine mediators, growth factors, receptors and binding proteins (insulin-like growth factor binding protein 5– ***IGFBP5***; *GHRL*; epidermal growth factor - ***EGF***) or related to digestion, nutrient uptake and transport (pepsinogen B and C; chitinase, acidic; lipoprotein lipase; solute carrier family 2, facilitated glucose transporter, member 4).

**Table 3:** Genes that were more expressed (*P*<0.05) in OXY mucosa, a priori selected for their relevance in the stomach, compared with PYL mucosa^1^.

		Gastric mucosa^2^		
Gene product	Gene name	OXY	PYL	SEM
*Acid secretion and pH homeostasis*				
H^+^/K^+^ Atpase α	*ATP4A*	12.1	4.6	0.09
Anion exchanger 2	*SLC4A2*	10.0	7.9	0.12
*Ion and water channels*				
Potassium voltage-gated channel, Isk-related family, member 2	*KCNE2*	10.2	3.9	0.20
Potassium inwardly rectifying channel, subfamily J, member 13	*KCNJ13*	8.0	3.3	0.27
Potassium inwardly rectifying channel, subfamily J, member 15	*KCNJ15*	8.8	3.7	0.17
Chloride intracellular channel 6	*CLIC6*	10.7	4.0	0.14
Aquaporin 4	*AQP4*	9.5	3.0	0.26
*Endocrine mediators, growth factors, receptors and binding proteins*				
Insulin-like growth factor binding protein 5	*IGFBP5*	10.1	8.5	0.25
Ghrelin/Obestatin Prepropeptide	*GHRL*	9.9	7.8	0.38
Epidermal growth factor	*EGF*	9.4	5.1	0.41
*Digestive enzymes, nutrient uptake and transport*				
Pepsinogen B	*PGB*	11.9	10.4	0.13
Pepsinogen C	*PGC*	12.2	11.3	0.14
Chitinase, acidic	*CHIA*	12.2	7.8	0.19
Lipoprotein lipase	*LPL*	9.8	7.6	0.46
Solute carrier family 2 (facilitated glucose transporter), member 4	*SLC2A4*	8.7	6.4	0.36
*Others*				
Alcohol dehydrogenase, iron containing, 1	*ADHFE1*	6.9	5.6	0.32

1 OXY  =  oxyntic mucosa; PYL  =  pyloric mucosa.

2 Mean values, expressed as log2 of intensity values.

Among the set of pre-selected genes, 16 genes were more expressed in PYL compared with OXY (Table 4). These genes were cell adhesion factors and regulators of tight junctions (Olfactomedin 4; Claudin 2 and 7) and were related to epithelial protection, immunity and detoxifying enzymes (lysozyme; polymeric immunoglobulin receptor; cytochrome P450, family 3, subfamily A, polypeptide 4– ***CYP3A46***; secretoglobin, family 1A, member 1 (uteroglobin) – ***SCGB1A1***); transcription factors (Meis homeobox 2; SRY (sex determining region Y)-box 21; leucine-rich repeat containing G protein-coupled receptor 5– ***LGR5***), endocrin mediators, growth factors, receptors and binding proteins (somatostatin; *GAST*), digestive enzymes, or nutrient transporters (gastric intrinsic factor), and others (aldo-keto reductase family 1, member C1; cysteine dioxygenase 1; adenylate kinase 5).

**Table 4:** Genes that were more expressed (*P*<0.05) in PYL mucosa, a priori selected for their relevance in the stomach, compared with OXY mucosa^1^.

			Gastric mucosa^2^					
Gene product	Gene name		OXY		PYL		SEM	
*Cell adhesion factors and tight junction regulation*								
Olfactomedin 4	*OLFM4*	4.8		9.4		0.69		
Claudin 7	*CLDN7*	5.8		9.1		0.33		
Claudin 2	*CLDN2*	5.0		7.5		0.48		
*Epithelial protection, immunity and detoxifying enzymes*								
Lysozyme	*LYZ*	10.3		11.7		0.22		
Polymeric immunoglobulin receptor	*PIGR*	8.3		10.4		0.23		
Cytochrome P450, family 3, subfamily A, polypeptide 4	*CYP3A46*	4.3		7.9		0.52		
Secretoglobin, family 1A, member 1 (uteroglobin)	*SCGB1A1*	3.8		8.0		0.57		
*Transcription factors*								
Meis homeobox 2	*MEIS2*	7.0		9.1		0.17		
SRY (sex determining region Y)-box 21	*SOX21*	5.5		6.5		0.18		
Leucine-rich repeat containing G protein-coupled receptor 5	*LGR5*	3.6		5.6		0.29		
*Endocrin mediators, growth factors, receptors and binding proteins*								
Somatostatin	*SST*	9.4		11.4		0.24		
Gastrin	*GAST*	5.5		10.6		0.30		
*Digestive enzymes, nutrient uptake and transport*								
Gastric intrinsic factor	*GIF*	10.0		11.4		0.37		
*Others*								
Aldo-keto reductase family 1, member C1	*AKR1C1*	4.3		8.0		0.48		
Cysteine dioxygenase 1	*CDO1*	5.1		7.9		0.50		
Adenylate Kinase 5	*AK5*	4.1		7.3		0.25		

1 OXY  =  oxyntic mucosa; PYL  =  pyloric mucosa.

2 Mean values, expressed as log2 of intensity values.

Other genes, characterizing the gastric mucosa versus the intestinal mucosae in mice, were not affected by the type of gastric mucosa (Table 5): ATPase, class V, type 10D– ***ATP10D***; carbonic anhydrase 2 & 11; gastrokine 1; protein disulfide isomerase (PDI) family A, 3 & 4; Mucin 1; glutathione peroxidase 1; glutathione S-transferase alpha 4; fatty acid binding protein 5; sodium iodide symporter, member 5; pancreatic amylase.

**Table 5:** Genes that did not differ for expression in OXY and PYL mucosa, a priori selected for their relevance in the stomach^1^.

			Gastric mucosa^2^					
Gene product	Gene name		OXY		PYL		SEM	
*Acid secretion and pH homeostasis*								
ATPase, class V, type 10D	*ATP10D*	6.3		6.5		0.66		
Carbonic anhydrase 2	*CA2*	11.8		11.6		0.11		
Carbonic anhydrase 11	*CA11*	5.4		6.0		0.30		
*Epithelial protection and detoxifying enzymes*								
Gastrokine 1	*GKN1*	11.8		11.9		0.44		
PDI family A, 3	*PDIA3*	10.9		10.9		0.23		
PDI family A, 4	*PDIA4*	9.8		9.8		0.16		
Mucin 1	*MUC1*	10.8		10.7		0.745		
Glutathione peroxidase 1	*GPX1*	9.1		9.3		0.45		
Glutathione S-transferase alpha 4	*GSTA4*	8.8		8.2		0.34		
*Endocrin mediators, growth factors, receptors and binding proteins*								
Fatty acid binding protein 5	*FABP5*	8.0		7.2		0.35		
*Digestive enzymes, nutrient uptake and transport*								
Solute carrier family 5 (sodium iodide symporter), member 5.	*SLC5A5*	7.8		8.4		0.27		
Pancreatic amylase	*AMY2*	5.7		5.1		0.33		
*Others*								
Cysteine sulfinic acid decarboxylase.	*CSAD*	5.8		5.4		0.18		

1 OXY  =  oxyntic mucosa; PYL  =  pyloric mucosa.

2 Mean values, expressed as log2 of intensity values.

The validation of the microarray data by quantitative real-time PCR analysis of four representative genes (*ATP4A*, *GAST*, *GHRL*, *PIGR*) is reported in table 6. The variations for the two different mucosae were confirmed for all the genes by the quantitative real-time PCR analysis.

**Table 6:** Validation of the microarray data by quantitative real-time PCR (qRT-PCR) analysis of four representative genes.

	Real Time RT-PCR^1^			Microarray^1^		
Gene name^2^	OXY	PYL	SEM^3^	OXY	PYL	SEM^3^
*ATP4A*	120.6	0.03	12.4	33.7	0.2	2.3
*GAST*	0.2	316.2	65.6	0.3	15.9	2.6
*GHRL*	12.84	4.2	2.6	7.5	2.6	1.1
*PIGR*	2.0	18.6	3.9	2.6	12.6	1.7

1 Values normalized for *hydroxymethylbilane synthase* and *ribosomal protein L4* gene expression.

2 *ATP4A*, H+/K+ Atpase α; *GAST*, gastrin; *GHRL*, ghrelin/obestatin prepropeptide; *PIGR*, polymeric immunoglobulin receptor.

3 All gene values differed for the different mucosae inside each analysis method (*P*<0.05).

## Discussion

### Expression in oxyntic mucosa

As expected, the comparison of OXY with PYL mucosa revealed a greater differentiation of OXY due to the presence of parietal cells responsible for the gastric hydrochloric acid secretion into the lumen of the stomach. This finding implies the powerful activation of H^+^/K^+^-ATPase to transport H^+^ across the apical membrane of parietal cells and explains the differential enrichment of genes collected in the pathway HYDROGEN ION TRANSMEMBRANE TRANSPORTER ACTIVITY. The enrichment of many MITOCHONDRIAL genes and pathways involved in membranes activity, oxidative phosphorylation and respiratory chain show the high ATP production and the massive use of energy primarily for proton pumping in oxyntic paretial cells. Interestingly, the gene for nicotinamide nucleotide transhydrogenase was enriched in OXY and present in both of these groups of genes and its activity is likely related to ATP biosynthesis [10] or free radical detoxification [11] in the cell. The hydrochloric acid secretion requires the exchange of bicarbonate for chloride ions; anion exchanger 2 contributes to basolateral membrane HCO3- transport [12]. Its observed gene upgrade confirms the relevance of this transporter for the ionic balance in the oxyntic mucosa, in agreement with observations of knock-out mice for this gene [13].

Class V *ATP10D*, which encodes phospholipid-translocating ATPase, was enriched in both in OXY and PYL mucosae while previously it has been shown to be localized in murine parietal cells [2]. However, the similar gene expression observed in our survey for OXY and PYL may indicate that this enzyme involved in the phospholipid translocation may be not only related to H^+^/K^+^+-ATPase membranes in parietal cells but also to the formation or reorganization of cellular or intracellular membranes or vesicular trafficking in the PYL [14]. Carbonic anhydrases catalyze the bidirectional conversion of carbon dioxide and water to bicarbonate and protons required for acid secretion. The enrichment in the transcripts for carbonic anhydrase 2 (cytosolic) and 11 (catalytic), which was observed in gastric corpus of mice in confront with intestinal segments [2], is observed in both gastric mucosae here. This result suggests that, beside the involvement of acid secretion, these carbonic anhydrases also serve to maintain the mucus-bicarbonate barrier by the mucus-producing epithelial cells in both gastric areas.

Regulation of the K^+^ balance in gastric surface cells and parietal cells is also relevant. Several genes transcribing for K^+^ channels were more strongly expressed in OXY than in PYL. *KCNE2*, which was the most expressed in parietal cells, in mice is associated with potassium the voltage-gated channel, KQT-like subfamily, member 1 (***KCNQ1***), forming an heterodimeric potassium channel [15]. The control of K^+^ fluxes to cytosol by this complex has relevance for acid secretion and is independent of H^+^/K^+^-ATPase activity in mice [15]. KCNQ1 was not present on our microarray chip. Therefore no data are available. However, KCNE2, which forms a complex with KCNQ1 to provide K-efflux for acid secretion in stimulated parietal cells [15] was highly expressed in OXY in our analysis confirming similar results in other species. In mice, He et al. [16] revealed the relevance of *KCNJ15* transcript in OXY and *KCNJ15* product in parietal cells, where it is stimulated by acid secretion and can cooperate with *KCNQ1*. *KCNJ15* is also present in chief cells, but not in mucous neck cells. A gene expression study of isolated parietal cells revealed that *KCNJ13* is present in significant amounts but shows the same or lower expression levels compared with whole gastric epithelium [17]. The relevance of total fluid excretion in OXY is finally outlined by the greater activation of *CLIC6*, which presumably creates a chloride ion gradient for water movement in parietal cells [18], in connection with water-selective channels like Aquaporin 4 in the plasma membranes to secrete water in the lumen and produce a more fluid bolus. Finally, these processes require the timely provision of energy substrates, which is evidenced by the increased transcription for genes related to lipolytic activity (*LPL*) and glucose transport (*SLC2A4*) in OXY compared with PYL. Nevertheless, the similar gene expression of *FABP5* in both gastric tissues may indicate that in PYL there is also an important need for fatty acids uptake, as has been evidenced for OXY in comparison with intestinal tissues in mice [2].

We also found that OXY shows increased expression of a gene related to digestion, the acidic chitinase (*CHIA*), which has already been identified in the stomach of other species, although with a variable degree of expression [19]. The activity of CHIA may be addressed to chitin-containing feeds (as may be also true for wild boar), to insects and other live organisms and be favored by the peculiar pH in OXY, which explains also the reduced gene expression in PYL.

Our results showed that OXY is more specialized for the control of feeding behavior given by the greater expression of genes of the FEEDING_BEHAVIOR group, where specifically ghrelin was enriched. Other endocrine mediators were found out to be more specifically involved in growth; EGF has a regulatory function on H^+^/K^+^-ATPase activity in parietal cells [20], thus its greater gene expression in OXY may be related to a paracrine control based on this growth factor that is not present in PYL. It is known that EGF expression and secretion in the stomach is regulated by capsaicin sensitive peripheral neurons and results in cytoprotective and antiulcerogenic activity together with the increase of CGRP and NO release [21], [22]. Furthermore, an upregulated gene expression of *IGFBP5* in OXY may be required for regulating cellular growth, differentiation and turnover in parietal cells [23].

*GPX1* and *GSTA4*, which were enriched in the stomach compared with the intestine [2], were not differentially expressed between OXY and PYL, supporting the detoxification role for all mucosa regions against reactive oxygen species [24] and xenobiotics [25]. Mucus is also important for the protection of the stomach, which is supported by the similar expression of the mucin 1 gene in OXY and PYL.

### Expression in pyloric mucosa

Other relevant gastric control mechanisms are also resident in PYL, including greater expression for GAST and its paracrine negative regulator, somatostatin, released from G-cells and D-cells, respectively. However, in PYL, all of the gastric content is forced to pass toward the intestine. Thus, the significant expression of genes of the LYMPHOCYTE ACTIVATION group in PYL is not surprising. It is furthermore reasonable to find that some proteins involved in mucosal defense such as antibacterial lysozymes and polymeric immunoglobulin receptors, required for IgA transepithelial basal-to-apical transport to the epithelial surface, were more expressed in PYL. The greater gene expression of polymeric immunoglobulin receptor in PYL versus OXY is consistent with previous observations using pigs at different ages [9].Our data reveal that other defense genes encoding for for protein disulfide isomerases (**PDI**) *PDIA3* and *PDIA4* are presumably not only highly expressed in chief cells, as reported previously [26], but also in PYL. *PDIA3* and *PDIA4* are involved in protein folding in rough endoplasmic reticulum and reported to be related to various function, in particular the assembly of major histocompatibility complex class I [27] and redox homeostasis [28], respectively.

It is also worth noting that the genes for the xenobiotic metabolizing cytochrome P450 enzyme, *CYP3A46* [29] and a secretoglobin (*SCGB1A1*), were more expressed in PYL. *CYP3A46* may have relevance for protecting the gut against T-2 toxin [30], the mold byproduct of *Fusarium* spp fungus, that, among other effects, causes vomit. Interestingly, SCGB1A1 is known for its anti-inflammatory properties and for the predominant localization in Clara cells of distal conducting pulmonary airways [31], [32]. Finally, the upregulation of genes related to lymphocyte activation may be also linked to the second genes set upregulated in PYL, LIPID_RAFTS. In fact, lipid rafts are cholesterol-rich microdomains of the plasma membrane known to be also involved in the activation of cytokine signaling [33] and T lymphocytes differentiation [34].

PYL mucosa also shows greater expression of some relevant genes related to the barrier defense of the mucosa: claudin 7 is, for example, necessary for the epithelial barrier integrity and to avoid bacterial translocation [35]. Furthermore, PYL seems to be better equipped than OXY to sustain a much greater turnover rate [36] and may be related to the constant mechanical stress caused by the passage of the feed bolus. This hypothesis is supported by the greater gene expression of the marker of gastro-intestinal stem cells *LGR5* [37]. Conversely, *GKN1*, another gene that is more involved in the renewal of gastric epithelium [38] compared with intestinal epithelium [2], was similarly expressed in both gastric areas, confirming that it is in general relevant to the replication of gastric surface mucous cells [37].

In conclusion, OXY and PYL mucosae show high expression of genes other than known functional genes related to hydrochloric acid and gastrin secretion. In general, the data indicate that OXY has a higher specialization than the PYL, useful for new marker detections. The pylorus expressed some gene transcripts that may merit additional studies, particularly those related to mucosal defense function. In addition, the research suggests that several genes are shared between OXY and PYL. hese new observations should be addressed in further studies considering the different compartments of the stomach separately, as is usually the case for the intestine, to reveal novel functions.
